# Postprimäre frühelektive Totalprothetik bei schweren Frakturen des oberen Sprunggelenks

**DOI:** 10.1007/s00113-022-01252-2

**Published:** 2022-11-22

**Authors:** Patrick Pflüger, Carsten Schlickewei, Alexej Barg, Victor Valderrabano

**Affiliations:** 1 Technische Universität München, München, Deutschland; 2 Klinik und Poliklinik für Unfallchirurgie und Orthopädie, Universitätsklinikum Hamburg-Eppendorf, Hamburg, Deutschland; 3 SWISS ORTHO CENTER, Professor University of Basel, Swiss Medical Network, Schmerzklinik Basel, Hirschgässlein 15, 4010 Basel, Schweiz

**Keywords:** Prothesendesign, Trauma, Ältere Patienten, Arthrose, Untere Extremität, Prosthesis design, Trauma, Aged, Osteoarthritis, Lower extremity

## Abstract

Die Versorgung des oberen Sprunggelenks (OSG) mithilfe einer Totalprothese (TP) ist heutzutage eine etablierte und sichere operative Therapie der Arthrose am OSG. Moderne Implantate haben geringe Revisionsraten und sind der Arthrodese des Sprunggelenks hinsichtlich des von Patienten berichteten Ergebnisses überlegen. Der Stellenwert der primären endoprothetischen Versorgung von Frakturen am OSG ist unklar. Aktuell finden sich diesbezüglich in der Literatur keine Studien. In Anbetracht der steigenden Fallzahlen instabiler Sprunggelenkfrakturen, insbesondere bei älteren Patienten und aufgrund wachsender funktioneller Ansprüche auch bis ins hohe Lebensalter, gilt es zukünftig beim Management dieser Frakturen auch eine endoprothetische Versorgung zu berücksichtigen. Klinische Studien sind notwendig, um die Versorgung von Frakturen des OSG mithilfe einer TP zu evaluieren.

## Abstract (en)

Treatment of the ankle joint with total ankle arthroplasty (TAA) is an established and safe surgical treatment for osteoarthritis of the ankle. Modern implants have low revision rates and are superior to ankle arthrodesis in terms of patient-reported outcomes. The importance of primary TAA treatment of ankle fractures is unclear. There are currently no studies in the literature on this topic. In view of the increasing number of cases of unstable ankle fractures, especially in older patients and with increasing functional demands also in old age, it is important to consider TAA in the management of these fractures in the future. Clinical studies are necessary to evaluate the treatment of ankle fractures using TAA.

Die Versorgung des oberen Sprunggelenks (OSG) mithilfe einer Totalprothese (TP) ist heutzutage eine etablierte operative Therapie der Arthrose mit guten bis sehr guten Langzeitergebnissen. In der Literatur finden sich jedoch zur primären endoprothetischen Versorgung von Frakturen am OSG aktuell keine Studien. In Anbetracht steigender Fallzahlen, insbesondere von instabilen Sprunggelenkfrakturen, und des wachsenden funktionellen Anspruchs bis ins hohe Lebensalter gilt es zukünftig, beim Management dieser Verletzungen auch die Option einer endoprothetischen Versorgung zu berücksichtigen.

## Geschichte der Totalendoprothetik und Implantatdesign

In den 1970er-Jahren berichteten erstmals Lord und Marrotte über eine Fallserie von 25 TP des OSG [22]. In den folgenden Jahren wurden weitere Fallserien unterschiedlich designter TP des OSG durch diverse Arbeitsgruppen veröffentlicht [11]. Alle Studien berichteten über vergleichbar schlechte Ergebnisse mit hohen Komplikationsraten [11], wobei insbesondere aseptische Lockerungen bei den ersten Implantaten in bis zu 90 % der Fälle nachgewiesen wurden [3].

Die meisten OSG-TP der ersten Generation, die in den 1970er- und frühen 1980er-Jahren eingesetzt wurden, waren zementierte und achsgeführte Zweikomponentenimplantate [4]. Um die mit der Prothesenkonfiguration assoziierten Komplikationsraten zu senken, wurden das Design sowie die Operationstechnik der OSG-TP stetig weiterentwickelt [4]. In den 1990er-Jahren wurden vornehmlich OSG-TP mit 3 Komponenten und zementfreier Implantation eingesetzt [14]. Heutzutage hat die OSG-Totalprothetik die Generation V erreicht [16]. Die modernen primären OSG-TP werden meist über einen anterioren Zugang eingebracht, sind zementfrei und verfügen über ein Polyethylen-Inlay. Die Kunststoffkomponenten lassen sich weiter in fixierte („fixed bearing“) und mobile („mobile bearing“) Inlays unterteilen [4]. Moderne Implantate bieten den Vorteil, die Knochenresektion durch das anatomisch präformierte Design möglichst gering zu halten [39]. Bei fortgeschrittenen Deformitäten oder im Revisionsfall stehen talar- und tibiaseitig Revisionsprothesenkomponenten mit entsprechend größerer Knochenverankerung zur Verfügung. Achsgeführte Systeme, wie beispielsweise am Kniegelenk, werden aufgrund der schlechten Ergebnisse nicht eingesetzt [4].

## Indikationen und aktuelle Ergebnisse

Die häufigste Indikation zur Implantation einer OSG-TP ist die fortgeschrittene, posttraumatische Arthrose des OSG [4, 37]. Bei den Betroffenen ist die Versorgung mithilfe einer OSG-TP von der American Orthopaedic Foot & Ankle Society (AOFAS) und der British Orthopaedic Foot & Ankle Society (BOFAS) als operative Standardbehandlung deklariert [1, 31].

Die Revisionsraten moderner OSG-TP betragen ungefähr 4–8 % innerhalb der ersten 5 Jahre

Darüber hinaus kann eine Prothesenversorgung des OSG als Revisionseingriff bei Patienten mit schmerzhafter Non- oder Malunion nach erfolgter Arthrodese des Sprunggelenks durchgeführt werden [13]. Die aktuellen Studien verzeichnen gute bis sehr gute Langzeitergebnisse mit nachhaltiger Verbesserung der Sprunggelenksfunktion sowie Prothesenstandzeiten von 82 % nach 15 bis 20 Jahren [4, 8, 19, 21, 40]. Insbesondere im Vergleich zur operativen Alternative, der Arthrodese des OSG, profitieren die Patienten hinsichtlich des selbstberichteten Ergebnisses signifikant vom endoprothetischen Ersatz [5, 10, 29]. Durch eine Versorgung mit einer OSG-TP kann die Gelenkfunktion wiedergeherstellt und den Patienten die Wiederaufnahme einer sportlichen Aktivität ermöglicht werden [2, 38]. Da die posttraumatische OSG-Arthrose meist jüngere, aktive Patienten betrifft, ist die endoprothetische Versorgung der Arthrodese in diesen Fällen überlegen [2].

Die Revisionsraten von OSG-Totalprothesen sind vergleichbar mit denen nach einer Arthrodese des Sprunggelenks und liegen bei den modernen Implantaten bei ungefähr 4–8 % innerhalb der ersten 5 Jahren [7, 26]. Im Vergleich zum endoprothetischen Hüft- oder Kniegelenkersatz ist die Revisionsrate bei OSG-TP somit noch etwas höher [20]. Die häufigsten Ursachen für ein Versagen der Sprunggelenkprothesen sind aseptische Lockerungen der tibialen und/oder talaren Komponenten, anhaltende Schmerzen und septische Lockerungen [28].

## Frakturen des oberen Sprunggelenks

### Epidemiologie

Frakturen des Sprunggelenks gehören beim Erwachsenen zu den häufigsten Frakturen und erreichen Inzidenzwerte von 168/100.000 und Jahr [9]. Die Epidemiologie der OSG-Frakturen weist 2 Höhepunkte auf. Während OSG-Frakturen bei Männern in der Adoleszenz oder im frühen Erwachsenenalter auftreten, häufen sich diese bei Frauen ab dem 40. Lebensjahr [9, 30, 35]. Die Verletzung bei Männern ist überwiegend auf ein Trauma mit hoher Energie zurückzuführen, dagegen werden OSG-Verletzungen bei Frauen hauptsächlich durch Traumata mit niedriger Energie verursacht [6, 17].

Insgesamt betrachtet sind unimalleolare Frakturen am häufigsten, gefolgt von bi- und trimalleolaren Frakturen. In den letzten Jahren ist jedoch eine Zunahme von multimalleolaren Sprunggelenkfrakturen v. a. bei älteren Frauen zu beobachten (OSG-Frakturen aufgrund von Osteopenie und Osteoporose; [6, 9]). Einen Spezialfall stellen Pilonfrakturen dar, welche die distale tibiale Gelenkfläche betreffen und typischerweise Folge eines Hochenergietraumas sind [23].

### Management und Outcome

Instabile und dislozierte Frakturen des OSG werden i. Allg. mit guten Ergebnissen operativ versorgt. Das Outcome ist jedoch maßgeblich von der Frakturkonfiguration, den Begleitverletzungen und den Nebenerkrankungen des Patienten abhängig [32, 33]. Die operative Versorgung insbesondere von Sprunggelenkfrakturen, die den posterioren Malleolus betreffen und/oder eine begleitende Syndesmosen- und Knorpelverletzungen aufweisen, kann mit schlechteren Ergebnissen einhergehen [25, 32, 36].

Eine Fraktur ist die häufigste Ursache für die Entwicklung einer OSG-Arthrose

Es gilt zu bedenken, dass ein Trauma die häufigste Ursache für die Entwicklung einer Arthrose des OSG ist und dies, insbesondere im jüngeren Patientenkollektiv, nichtzufriedenstellende Langzeitergebnisse bedingen kann [33, 37]. Die Latenzzeit zwischen OSG-Fraktur und OSG-Arthrose kann von einem Jahr bis hin zu 52 Jahren stark variieren; der Durchschnitt beträgt 20,9 Jahre [15]. Eine kurze Latenzzeit von einem bis 2 Jahren ist häufig mit einer intraartikulären mehrfragmentären Fraktur, einem High-Energy-Trauma mit postprimärer Apoptose der Chondroblasten, mit Osteopenie oder Osteoporose sowie mit patientenabhängigen Negativfaktoren (Begleiterkrankungen) vergesellschaftet [15]. Beim Management von Sprunggelenkfrakturen des älteren Patienten gilt es oft vorhandene Nebenerkrankungen wie Diabetes mellitus, Osteoporose und Störungen des Herz-Kreislauf-Systems zu beachten [24]. Insbesondere Weichteilkomplikationen treten bei diesen Patienten vermehrt nach offener Reposition und interner Osteosynthese auf, weshalb immer häufiger intramedulläre Implantate zur Frakturversorgung verwendet werden [36].

Neben den patientenspezifischen Faktoren kann insbesondere der Unfallmechanismus (High-Energy-Trauma) zu erheblichen Weichteilverletzungen führen. So erfordern Pilonfrakturen infolge des Hochenergietraumas oftmals ein multidisziplinäres Management. Eine anatomische Wiederherstellung des Gelenks mithilfe der offenen Reposition ist in solchen Fällen oft nur eingeschränkt möglich, und es muss auf externe Fixationsmöglichkeiten zurückgegriffen werden [23].

## Fallbeispiel

Wie an der Schulter, der Hüfte und teils am Knie kann die OSG-TP auch eine Rolle spielen in der Versorgung von intraartikulären oder schweren Frakturen des OSG, die frühzeitig in der endgradigen schmerzhaften OSG-Arthrose enden: Fälle mit kurzer Latenzzeit zwischen Fraktur und OSG-Arthrose. Die postprimäre frühelektive OSG-TP kann eine gute Alternative zur OSG-Arthrodese darstellen, um eine Schmerzbehandlung mit Funktionserhalt am OSG und eine Verbesserung der Lebensqualität zu erzielen.

**Figure 1:**
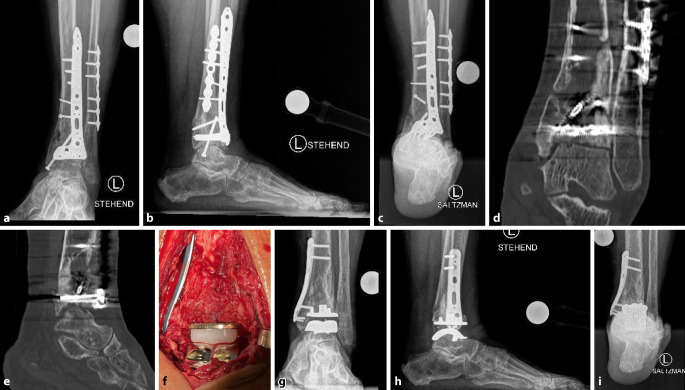

Beispielhaft wird im Folgenden der Fall einer 78-jährigen aktiven Patientin mit einer posttraumatischen, chronisch-schmerzhaften, zystischen Früharthrose des linksseitigen OSG, einer Knochennekrose im OSG-Plafond sowie einem medialen und ventralen Tibia-Defekt vorgestellt (Abb. 1). Bei Z. n. primärer Osteosynthese einer distalen Unterschenkel‑/Pilonfraktur 14 Monate zuvor betrugen der Schmerzscore auf der visuellen Analogskala (VAS) 8 und der AOFAS-Ankle-Funktionsscore 36 Punkte. Die Rekonstruktion mithilfe der postprimären frühelektiven OSG-TP wurde wie folgt vorgenommen: Osteosynthesematerialentfernung, Implantation des OSG-TP-Systems mit mobilem Inlay (Vantage Total Ankle Arthroplasty Mobile; Fa. Exactech, Gainesville, FL, USA) sowie mediale und ventrale Tibia-Knochenaufbauplastik mit supramalleolärer Stabilisierung durch eine winkelstabile mediale Tibia-Platte (Fa. Medartis, Basel, Schweiz). Bereits 3 Monate nach der Implantation ist die Patientin beschwerdefrei (VAS Schmerzscore 0) und erreicht einen AOFAS-Ankle-Funktionsscore von 100 Punkten.

## Diskussion und Ausblick

Eine primäre frakturendoprothetische Versorgung ist als erfolgreiche Therapie nur bei wenigen Gelenken etabliert. An der oberen Extremität ist die Implantation einer inversen Schulterprothese bei älteren Patienten mit komplexen Humeruskopffrakturen einer gelenkerhaltenden Rekonstruktion mithilfe der „open reduction internal fixation“ (ORIF) überlegen [34]. Im Bereich der unteren Extremität ist die Versorgung von Schenkelhalsfrakturen mithilfe von Hemi- oder Totalhüftendprothesen anzuführen. Die Indikation zur primären endoprothetischen Versorgung ist jedoch maßgeblich vom Patientenalter und vom funktionellen Anspruch abhängig [18].

Auch bei Frakturen des Kniegelenks existieren zur primären endoprothetischen Versorgung außer kleineren Fallserien keine Studien. Obwohl sich die Indikation nur auf ein ausgewähltes Patientenkollektiv beschränkt, sind die Komplikationsraten signifikant höher als bei der Implantation elektiver Knietotalprothesen. Darüber hinaus ist die operative Versorgung anspruchsvoll und setzt Kenntnisse der Revisionsendoprothetik voraus [27].

Die primäre frakturendoprothetische Versorgung bei schweren Frakturen des OSG stellt zurzeit keine etablierte Therapieoption dar, da die primäre Fixation der OSG-TP an den Frakturfragmenten schwierig ist. Es finden sich hierzu auch keine Studien in der aktuellen Literatur. Die Implantation von OSG-TP ist generell technisch anspruchsvoll, und die Lernkurve ist recht flach [4]. Schwere Frakturen des OSG betreffen entweder junge Patienten im Rahmen von Hochenergietraumen oder ältere Patienten mit osteoporotischem Knochen und relevanten Nebenerkrankungen. Bei jüngeren Patienten möchte man eine endoprothetische Versorgung mit notwendiger größerer ossärer Verankerung möglichst verhindern. Das akute Weichteiltrauma verursacht altersunabhängig, insbesondere nach offener operativer Versorgung, relevante Komplikationen. Eine aufwendige endoprothetische Versorgung mit Zusatzmaßnahmen, um die Stabilität wiederherzustellen, ist daher bei schweren Frakturen des OSG kritisch zu sehen.

Die postprimäre frühelektive OSG-TP kann in ausgewählten Fällen schwerer OSG-Frakturen sinnvoll sein

Eine endoprothetische Versorgung von OSG-Frakturen kann jedoch erfolgreich früh-elektiv bzw. postprimär nach Ausheilung der Frakturfragmente erfolgen. Mit einem ausreichenden „bone stock“ lassen sich durch den Einsatz der modernen OSG-TP bei geringem Operationstrauma eine gute bis sehr gute Funktion, Schmerzreduktion bis -aufhebung sowie schnelle Wiedererlangung der Beweglichkeit erzielen.

Die postprimär vorliegenden Befunde müssen gut und kritisch evaluiert werden, um das beste Ergebnis für den Patienten zu erzielen. Bei älteren Patienten mit einer Osteoporose, einem geringeren funktionellen Anspruch und einer instabilen Fraktur des OSG kann die Arthrodese des Sprunggelenks die bessere Therapiealternative darstellen [12]. Anders gestaltet sich dies bei sportlich aktiven Patienten mit einer Pilonfraktur. Hier gilt es, eine Weichteilkomplikation oder gar eine frakturbedingte Infektion bei der primären Osteosynthese durch ein geringes technisches Operationstrauma möglichst zu verhindern und die Funktion bestmöglich wiederherzustellen. Bei Entwicklung einer frühzeitigen posttraumatischen OSG-Arthrose kann im Verlauf eine früh-elektive, postprimäre OSG-TP-Versorgung durchgeführt werden.

## Fazit für die Praxis

- Totalprothesen des oberen Sprunggelenks (OSG-TP) haben gute Überlebenswahrscheinlichkeiten und sind eine etablierte operative Therapieoption zur Behandlung der endgradigen OSG-Arthrose.
- Die Versorgung mit einer modernen OSG-TP ist einer OSG-Arthrodese hinsichtlich des von Patienten berichteten Ergebnisses überlegen.
- Es gibt keine Evidenz bezüglich einer primären OSG-TP bei Frakturen des OSG.
- Eine postprimäre frühelektive OSG-TP kann in ausgewählten Fällen von schweren Frakturen des OSG, die in einer frühen Gelenkzerstörung enden, eine Therapieoption darstellen, um eine OSG-Arthrodese zu verhindern.
- Studien sind notwendig, um den Stellenwert der OSG-TP bei Frakturen des OSG zu evaluieren.
